# Cx43 mediates changes in myofibroblast contraction and collagen release in human amniotic membrane defects after trauma

**DOI:** 10.1038/s41598-021-94767-4

**Published:** 2021-08-18

**Authors:** Eleni Costa, Babatunde O. Okesola, Christopher Thrasivoulou, David L. Becker, Jan A. Deprest, Anna L. David, Tina T. Chowdhury

**Affiliations:** 1 Institute of Bioengineering, School of Engineering and Materials Science, Queen Mary University of London, Mile End Road, London, E1 4NS UK; 2 Department of Cell and Developmental Biology, University College London, Gower Street, London, WC1E 6BT UK; 3 Lee Kong Chian School of Medicine, Nanyang Technological University, 11 Mandalay Road, Singapore, Singapore; 4 Department of Obstetrics and Gynecology, University Hospitals Leuven, Leuven, Belgium; 5 Institute for Women’s Health, University College London, Medical School Building, Huntley Street, London, WC1E 6AU UK

**Keywords:** Trauma, Biomedical engineering

## Abstract

The wound healing capacity of the fetal membranes after spontaneous or iatrogenic membrane rupture is unclear. We examined the healing mechanisms in amniotic membrane (AM) defects after trauma. Traumatised human AM defects were cultured for 4 days. Markers for nuclear (DAPI), cell type (vimentin, αSMA) and healing (Cx43, TGFβ_1_, collagen) were examined by immunofluorescence (IMF) confocal microscopy, Second Harmonic Generation (SHG) imaging and RT-qPCR. After trauma, AMCs and myofibroblasts migrated to the AM wound edge. Within four days, αSMA expressing myofibroblasts showed abundant Cx43 localized in the cytoplasmic processes. The highly contractile spindle-shaped myofibroblasts were present in the defect site and released collagen. In contrast, AMCs expressed vimentin and formed Cx43 plaques between cells found in the outer edges of the wound. Whilst AMCs were absent in the defect site, αSMA expressing myofibroblasts continued to elongate and polarize the collagen fibres. Both TGFβ_1_ and Cx43 gene expression were significantly increased after trauma. Cx43 has differential effects on AM cell populations that increase cellularity, contraction and potentially migration to the wound edge resulting in collagen polarisation in the AM defect site. Establishing how Cx43 regulates AM cell function could be an approach to repair defects in the membranes after trauma.

## Introduction

Iatrogenic preterm premature rupture of the fetal membranes (PPROM) is a major complication after diagnostic or invasive fetoscopic interventions and is often associated with adverse perinatal outcome. In around 30% of fetal surgeries, the membranes separate leading to loss of tissue strength, amniotic fluid leakage and intrauterine infection^1–6^. In most cases, there is limited healing potential of the membranes after fetoscopic or open fetal surgery^6–11^. Women who rupture their membranes after amniocentesis deliver later in gestation and sometimes there is amniotic fluid reaccumulation^6^. Occasionally in iatrogenic membrane defects, there is evidence of a wound resealing effect rather than a healing mechanism such that the membranes slide apart, contract or reposition over the defect site and the fluid is unable to leak out^9–12^. A clinical solution to improve healing of the fetal membranes after fetal therapy or spontaneous rupture would therefore be highly desirable.

Fetal membrane wound healing has been observed preclinically, with the rate of wound healing dependent on the species (rodents vs rabbits), model system (e.g. tissue region, puncture device, defect size) and time for repair after injury (days vs months). Previous studies in swine, mice or rats reported spontaneous healing of fetal membrane defects sized between 0.47 and 4 mm in diameter a few days after injury^7, 8^. In mouse fetal membranes, the healing rate was dependent on the size of the defect created by the needle (20 or 26 gauge), with 100% closure in small wounds (0.47 mm) than large diameter (0.91 mm) wounds^7^. In mouse membranes, healing involved differentiation of amniotic epithelial cells (AECs) to a mesenchymal-like phenotype and macrophage cell recruitment from the amniotic fluid into the defect site^7^. This mechanism was absent in swine despite spontaneous healing of membrane defects up to 4 mm in diameter 44 days post laparotomy^8^. Limited healing after injury was also reported in fetal membrane defects from sheep, rhesus monkeys, humans and rabbits^10–14^. The differences in the healing response could be due to the increased vascular supply to swine and mouse fetal membranes which will enhance the rate of inflammation via IL-1β and TNFα at the ruptured site and enable granulation tissue formation and scarring^7, 8^. Whilst swine and mouse models have spontaneous membrane healing properties, they may not be suitable when translated for repairing defects in human fetal membranes.

The mechanism of healing involved proliferating AECs reported to lose cell to cell adhesion, apical-basal polarity and differentiated to amniotic mesenchymal cells (AMCs) and expressed high levels of vimentin^7, 15^. This intermediate protein is an established mediator of cell migration and wound repair, and in mouse membrane defects was reported to accelerate tissue repair mechanisms^7^. The release of growth factors like transforming growth factor subtype 1 (TGFβ_1_) by macrophages stimulates fibroblast-like cell differentiation to myofibroblasts and promote repair via α-smooth muscle actin (αSMA)^7, 16–19^. Embryonic wound healing involves the formation of an actomyosin ring at the wound margin, which enables purse-string contraction of the wound without activation of an inflammatory mechanism^20, 21^. We and others have shown that overexpression of Cx43 and gap junction plaque formation interferes with cell mobility leading to an absence of wound closure in chronic skin and AM wounds, probably through preventing the purse-string contraction mechanism^22–25^. Using a human AM defect model, myofibroblasts increased expression of cytoplasmic Cx43 and contractile function in the fibroblast layer. αSMA positive myofibroblasts were observed in the AM defect site and produce collagen fibres that polarized and potentially contracted the edges of the wound. Whilst AMCs expressed vimentin, the dense plaque formation of Cx43 found between cells was observed to prevent migration across the outer edges of the wound. The purse-contraction mechanism mediated by αSMA expressing myofibroblasts was observed four days after trauma with significant acceleration of cellularity in conjunction with collagen remodelling in an attempt to speed up wound closure in the human AM defect.

## Results

### Morphological and structural features of the human fetal membrane

**Figure 1:**
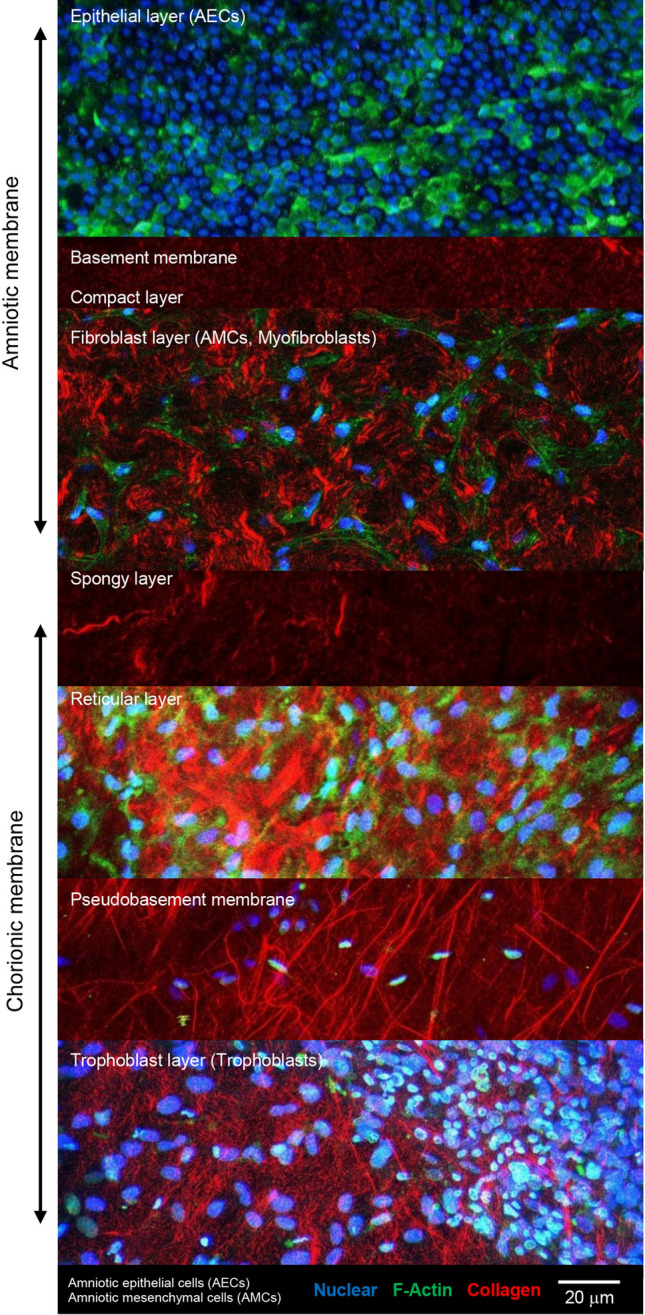
Features of the human fetal membrane. Differences in cell type and collagen distribution were found with tissue depth. IMF confocal microscopy showed a single layer of flattened AECs present in the epithelial layer (blue; green F-actin) in contrast to elongated AMCs and myofibroblasts (green) in the fibroblast layer. SHG imaging revealed a collagen fibrillar network (red) that interconnects the basement membrane in the AM with the compact and intermediate spongy layer. In the CM, the layers of the spongy, reticular and pseudo-basement membrane contain trophoblast cells (blue) and exhibit unique collagen characteristics. Blue (DAPI) and green (phalloidin) signals staining of F-actin detected by confocal imaging, respectively. Scale bar = 20 μM.

Figure 1 shows differences in cell morphology and collagen distribution in the AM compared to the CM. IMF confocal microscopy showed a single layer of flattened, cuboidal AECs present in the epithelial layer (blue) in contrast to elongated AMCs and myofibroblasts (green) in the fibroblast layer. SHG imaging revealed a collagen fibrillar network (red) that interconnects the basement membrane in the AM with the compact and intermediate spongy layer. In the CM, the layers of the spongy, reticular and pseudo-basement membrane contain trophoblast cells (blue) and exhibit unique collagen characteristics. Since membrane integrity and mechanics are enhanced by collagen organisation, further studies examined the effects of trauma in the AM.

### Cell phenotype and collagen changes in human AM defects after trauma

**Figure 2:**
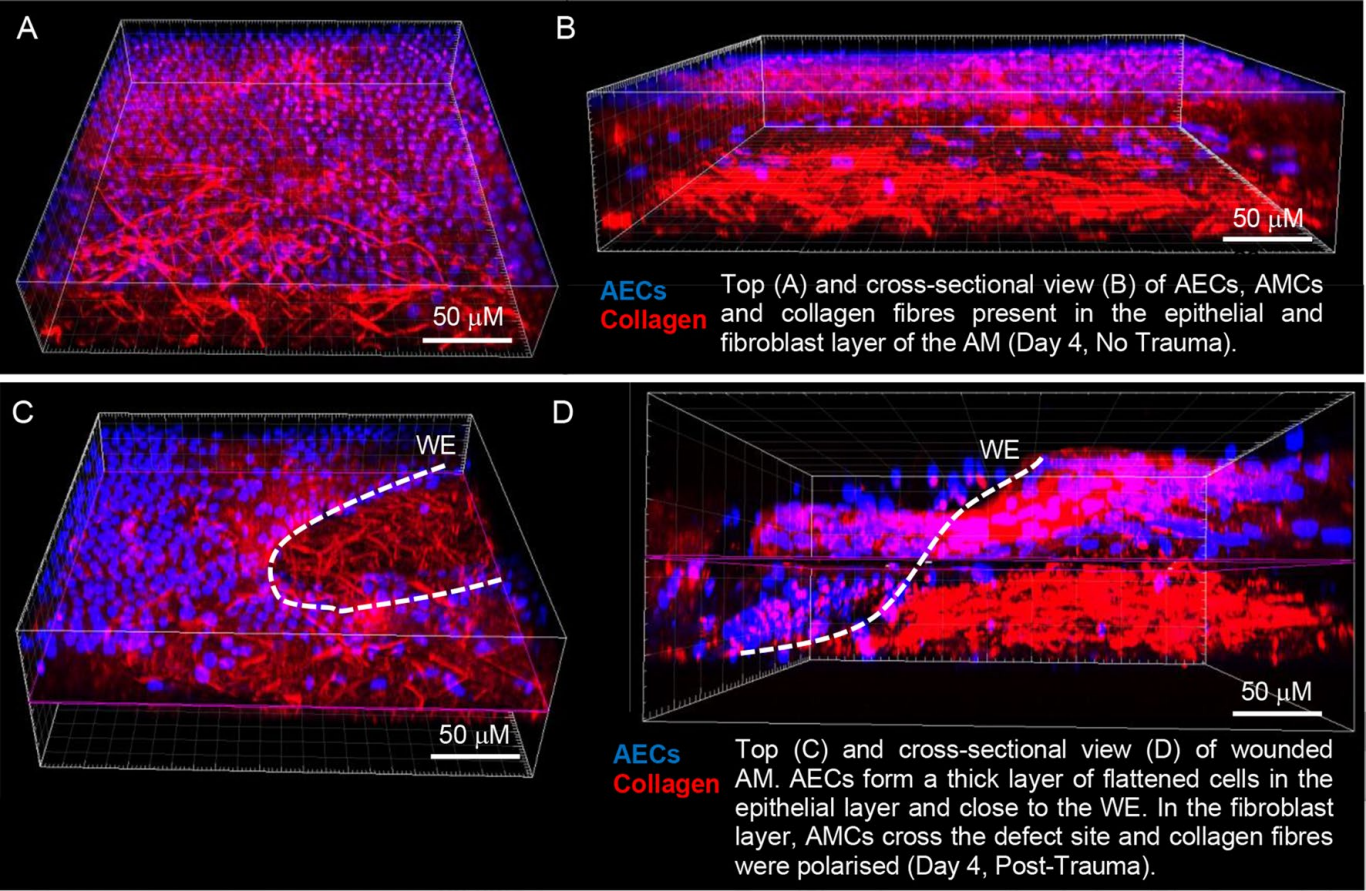
Morphological changes in human AM defects after trauma. In control AM specimens, we observed a layer of flattened AECs in the epithelial layer (**A**) and a dense region of collagen fibres in the fibroblast layer after four days of culture by IMF confocal microscopy and SHG imaging (**B**). After creation of a 0.8 mm defect in the AM specimen and culture for up to 4 days, we observed a population of highly contractile fibroblast-like cells that had crossed the defect site (**C**) with evidence of a greater intensity of the SHG signal close to the wound edge (WE) and polarization of collagen fibres (**D**). Blue signal is DAPI staining of nuclei detected by confocal imaging and red signals (collagen) were detected by SHG imaging. The dotted white lines show the border along the length of the WE in the AM specimen. Scale bar = 50 μM.

**Figure 3:**
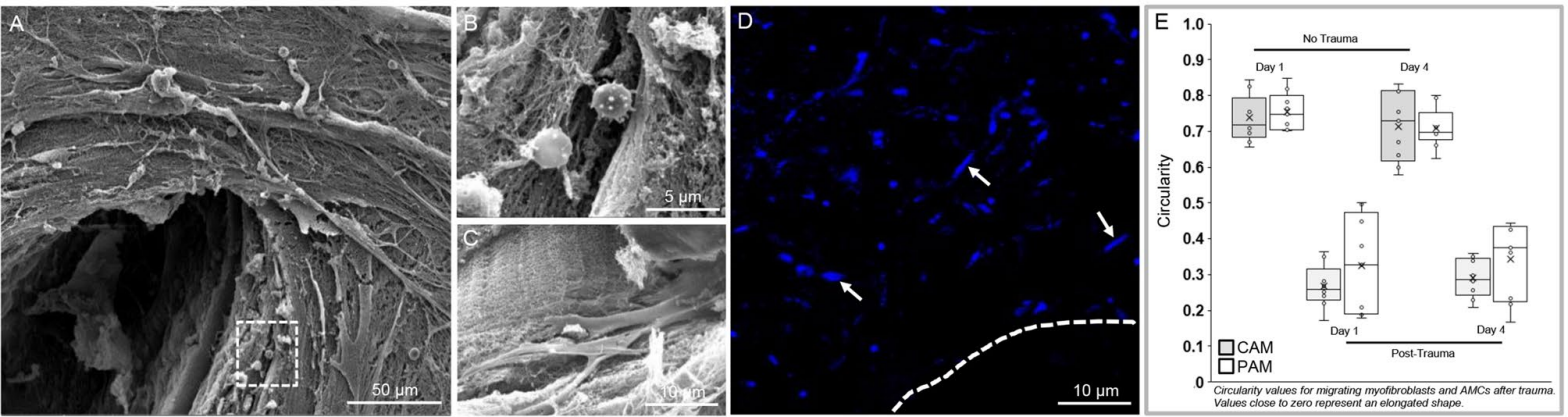
Characteristics of cell populations in wounded AM. Analysis by SEM showed increased cellularity of highly contractile cell populations that had migrated to the wound edge (**A**). At the defect site, we observed the presence of macrophages (**B**) and elongated spindle-like shape cells showing long cytoplasmic processes (**C**) and deformed nuclei (blue, **D**). After 4 days of trauma, cell circularity values were close to zero when compared to Day 1 and represent nuclear deformation in wounded PAM and CAM specimens (**E**). Box and whisker plots represent nine values taken from three donors.

In control AM specimens cultured for up to 4 days, we observed a layer of flattened, cuboidal AECs in the epithelial layer (Fig. 2A) and a dense region of polarized collagen fibres in the fibroblast layer by IMF confocal microscopy and SHG imaging (Fig. 2B), respectively. After creation of a 0.8 mm defect in the AM and culture of wounded specimens for up to 4 days, a population of cells appears to migrate into the defect site with evidence of a greater intensity of the SHG collagen signal across the defect site and close to the edges of the wound (Fig. 2C,D). SEM analysis of the migrating cell populations confirmed highly contractile AMC morphology with elongated cell bodies and cytoplasmic extensions (Fig. 3A–C). We also observed the presence of macrophage cell clusters at the edges of the wound (Fig. 3B). Nuclei and cytoplasmic polarization were tangential to the edges of the wound (Fig. 3D) indicating increased cell deformation and contraction after trauma (Fig. 3E).

### Differential αSMA and Cx43 protein expression by cell populations in the AM defect

**Figure 4:**
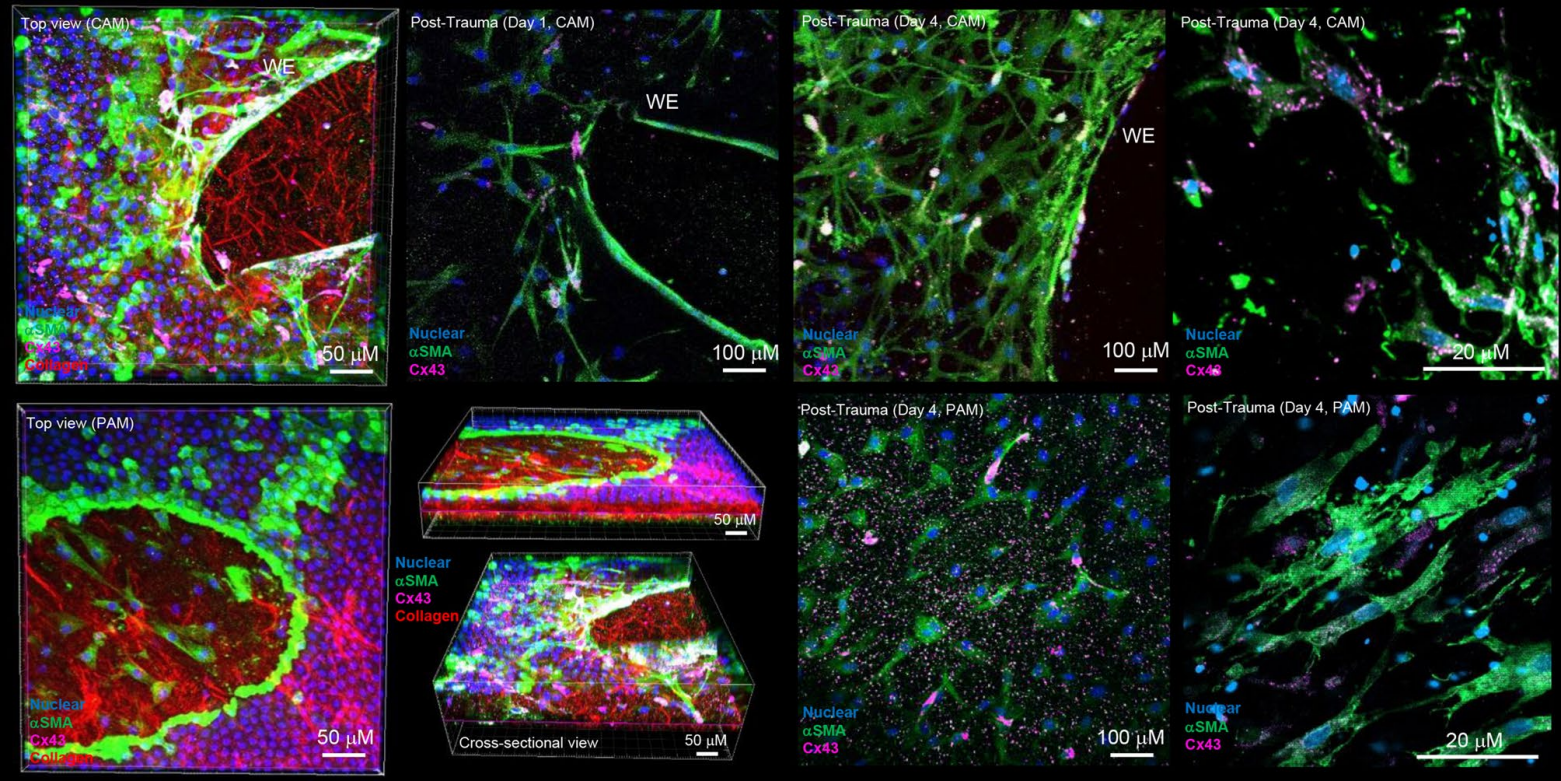
Cx43 expression by myofibroblasts in wounded AM defects. After trauma, we observed increased cellularity around the wound edge (WE) and the presence of αSMA expressing myofibroblasts that had migrated into the defect site and released collagen in CAM and PAM specimens (Top view). In the epithelial layer, AECs formed a thick layer around the WE and the cells did no not migrate into the defect site (cross-sectional view). In the fibroblast layer, the dense region of collagen fibres in the defect site were highly polarized and begin to contract the edges of the wound (Cross-sectional view). We observed punctate staining of Cx43 expression (pink) by myofibroblasts with cytoplasmic localization after four days of trauma (high magnification) in CAM and PAM defects. Blue (nuclear), green (αSMA), pink (Cx43) and red signals (collagen) were detected by confocal microscopy and SHG imaging, respectively. Scale bars = 20 μΜ and 50 μM.

Human AM specimens from cervix and placental regions showed multiple cell populations in the wounded tissue after trauma (Top view, Fig. 4). In the fibroblast layer, αSMA (green) expressing myofibroblasts increased expression of Cx43 (pink), facilitating contraction (Cross-sectional view, Fig. 4) and potential migration into the defect site (Supplementary Video). In the fibroblast layer, the αSMA expressing myofibroblasts and collagen fibres were highly polarized and begin to pull the edges of the wound in and across the defect site (Supplementary Video). In contrast, AECs formed a thick layer of flattened cells around the edges of the wound in the epithelial layer (Top View. Fig. 4). Cx43 protein expression was found to be localized in myofibroblast cytoplasmic bodies in wounded specimens (Fig. 4, Pink).

**Figure 5:**
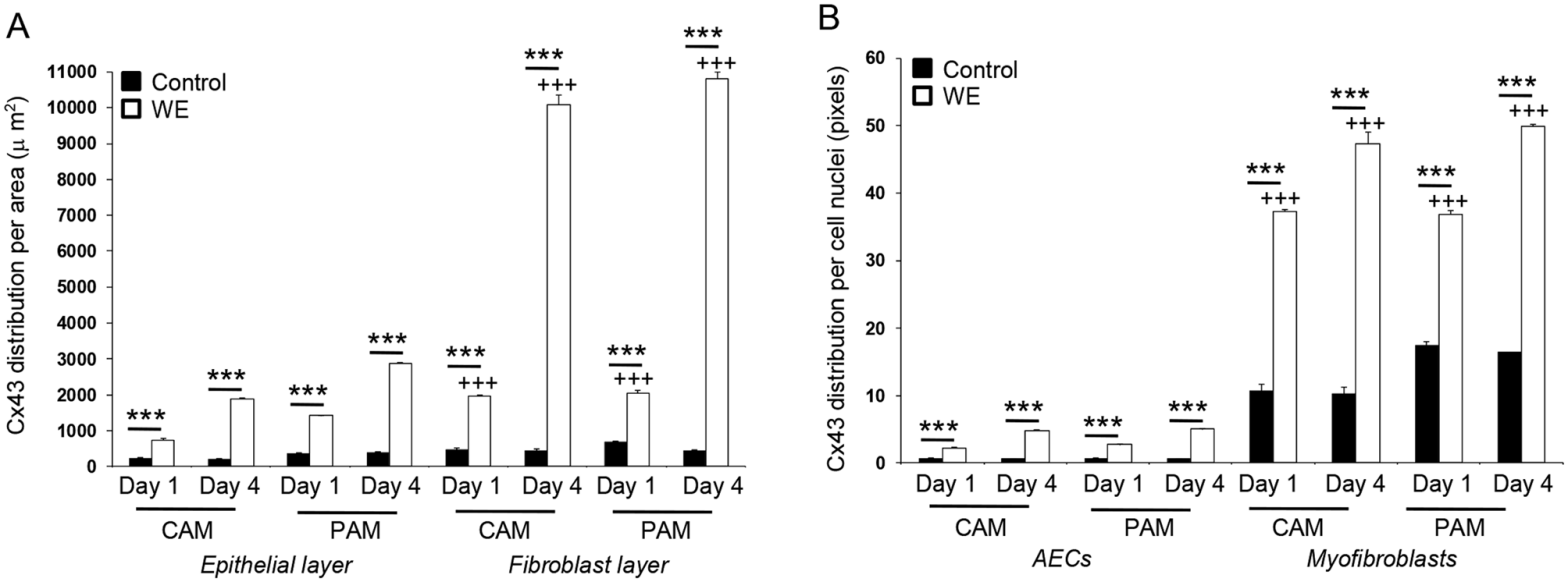
Cx43 protein expression in AM defects after trauma. The distribution of Cx43 was analysed per unit tissue area for comparisons between epithelial and fibroblast layer (**A**) and per AEC or myofibroblast cell nuclei (**B**) in control and wound edge (WE) specimens. In all cases, error bars represent the mean and SEM values for n = 9 replicates, where membranes were taken from three donors. Significant differences were found as indicated by ***p < 0.001. Comparisons between CAM or PAM specimens in the epithelial and fibroblast layer are indicated by  ^+++^ p < 0.001. All other comparisons were not statistically significant (not indicated).

Quantitative analysis of Cx43 protein distribution in the AM and per cell subpopulation are presented in Fig. 5. In control AM were low with values ranging from 205.5 to 397.8 μm^2^ in the epithelial layer and 441.2 to 698.7 μm^2^ in the fibroblast layer (Fig. 5A). After trauma, the levels of Cx43 protein expression significantly increased in wounded AM with maximal values after four days of trauma in the fibroblast layer for CAM (10,086.3 μm^2^) and PAM specimens (10,821.1 μm^2^) when compared to the wounded epithelial layer (< 2500 μm^2^) or control (< 250 μm^2^) AM specimens (Fig. 5A; all p < 0.001). In wounded AM, Cx43 pixel distribution was dependent on the cell type with lowest values found for AECs (0.62–0.74 pixels) in contrast to myofibroblasts (47.3–49.9 pixels) in CAM and PAM specimens (all p < 0.001, Fig. 5B). In contrast, values for Cx43 protein expression in controls were very low (< 0.2 pixels) when cultured for the same time period.

**Figure 6:**
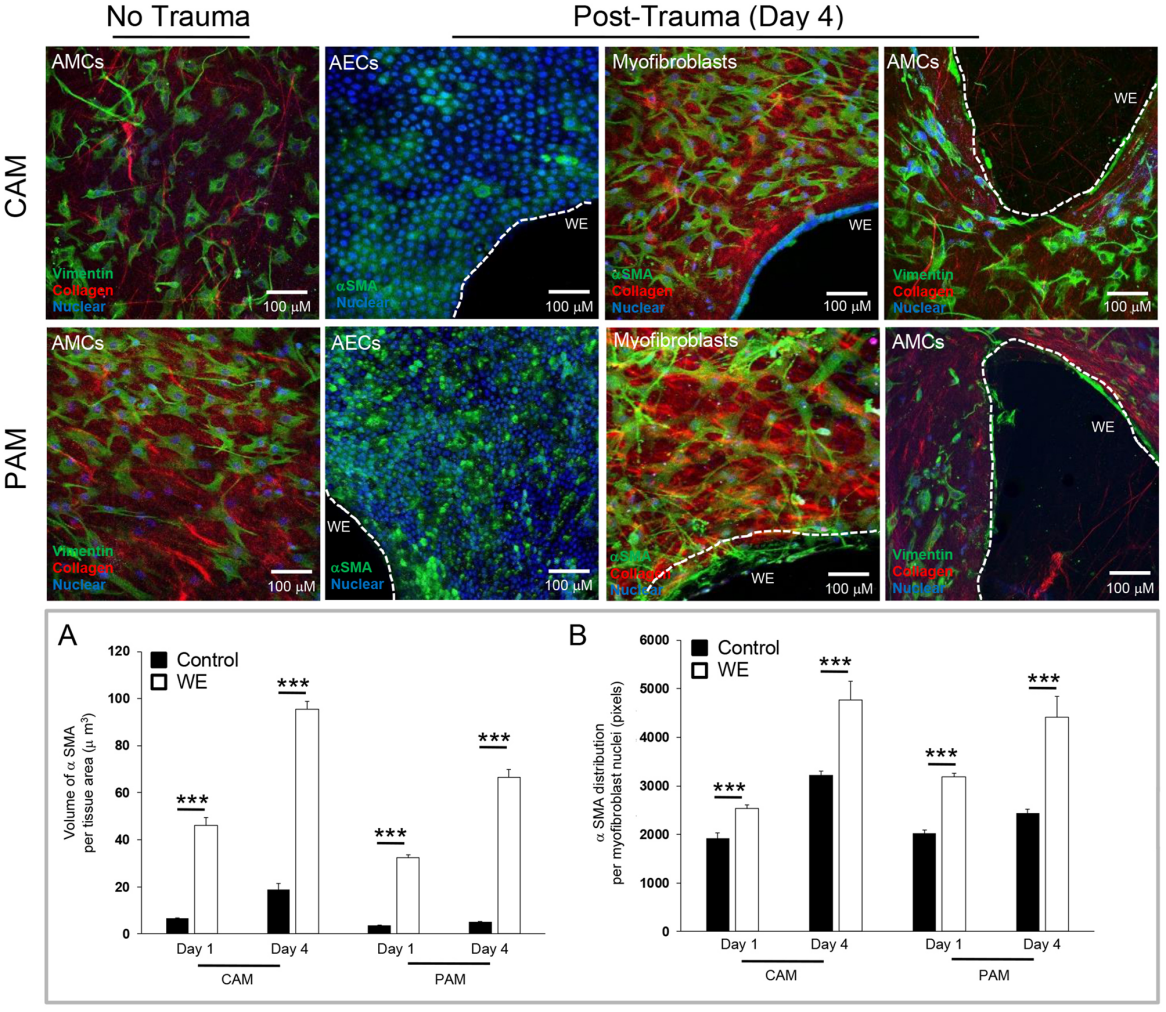
αSMA protein expression by myofibroblasts in AM defects after trauma. We observed abundant expression of αSMA by myofibroblasts when compared to AECs and AMCs (Top panel). Localisation of vimentin in stress fibres by AMCs showed increased contraction and migration to the wound edge (WE) but not into the defect site. Increased αSMA overexpression localized in stress fibres synthesized collagen fibres and polarization. Bottom panel quantifies the distribution of αSMA analysed per volume of tissue area in the fibroblast layer (**A**) and per myofibroblast cell nuclei (**B**) between control and wound edge (WE) specimens. In all cases, error bars represent the mean and SEM values for n = 9 replicates, where membranes were taken from three donors. Significant differences were found as indicated by ***p < 0.001. All other comparisons were not significant (not indicated).

Figure 6 compares αSMA protein expression in AECs with myofibroblasts and AMCs in the AM defect after trauma by IMF confocal microscopy. We observed minimal protein expression of αSMA in AECs in contrast to high expression in contractile myofibroblasts (Top panel, Fig. 6). In contrast, vimentin-positive AMCs did not express αSMA in wounded CAM and PAM specimens (Top panel, Fig. 6). In control CAM specimens, the volume of αSMA-expressing myofibroblasts was low with values approximately 6.45 μm^3^. After trauma, the values significantly increased to 46.1 μm^3^ (Day 1) and 95.6 μm^3^ (Day 4) in myofibroblasts (all p < 0.01, CAM) and were low in AMCs and AECs (< 0.2 μm^3^).

### Collagen organization in the AM defect after trauma

**Figure 7:**
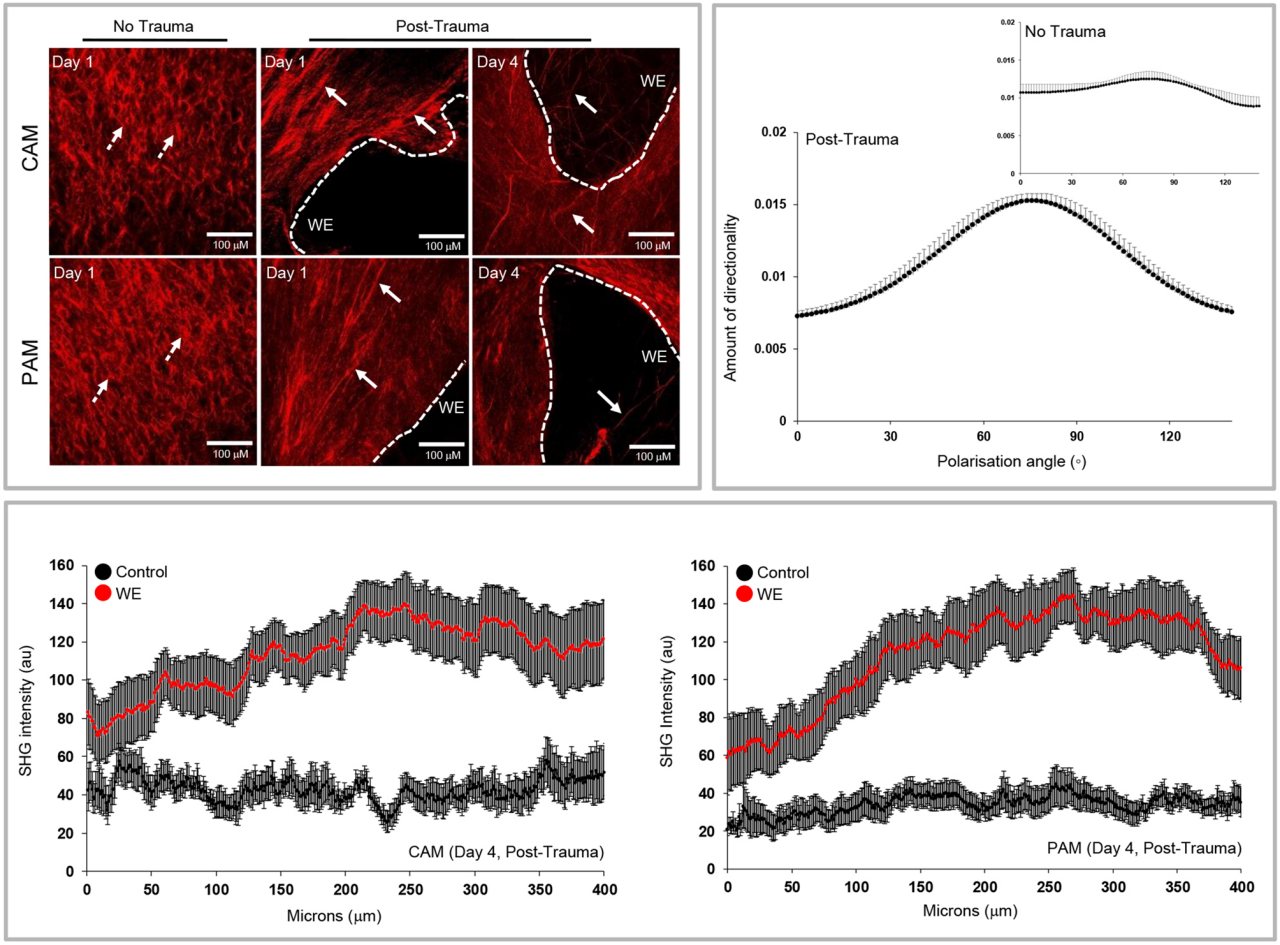
Collagen fibre organization in AM defects after trauma. SHG imaging of collagen (red) showed dense collagen fibre alignment tangential to the wound edge (WE) in CAM and PAM specimens but not in control specimens (Top panel). After trauma, there was a region of highly polarized collagen fibres aligned in the direction at around 90’C in wounded specimens. This intense organization was absent in control specimens. Quantification of collagen fibres revealed a significant increase in the SHG intensity values for WE specimens than controls (Bottom panel). The dotted white lines show the length of the WE in the AM. Scale bar = 100 µm.

Control and wound edge AM were examined by SHG imaging in the AM after 1 and 4 days of trauma (Fig. 7). Representative images in control AM appeared compact, irregular, and basket-like in the basement, intermediate and spongy layers (Top panel, Fig. 7). This arrangement appeared dense, elongated and highly aligned with evidence of a greater intensity of the SHG signal close to the wound edge of the AM and fibril secretion across the defect site (white arrows). Polarisation was maintained after 4 days of trauma (Top panel, Fig. 7). At the defect site, the direction of collagen fibre organization showed a region of highly polarized fibres at around 90 °C with a spread between 0 and 180 °C. After four days of trauma, the SHG values were greater in wounded specimens compared to controls (Bottom panel, Fig. 7).

### Trauma increased Cx43 and TGFβ1 gene expression in wounded AM

**Figure 8:**
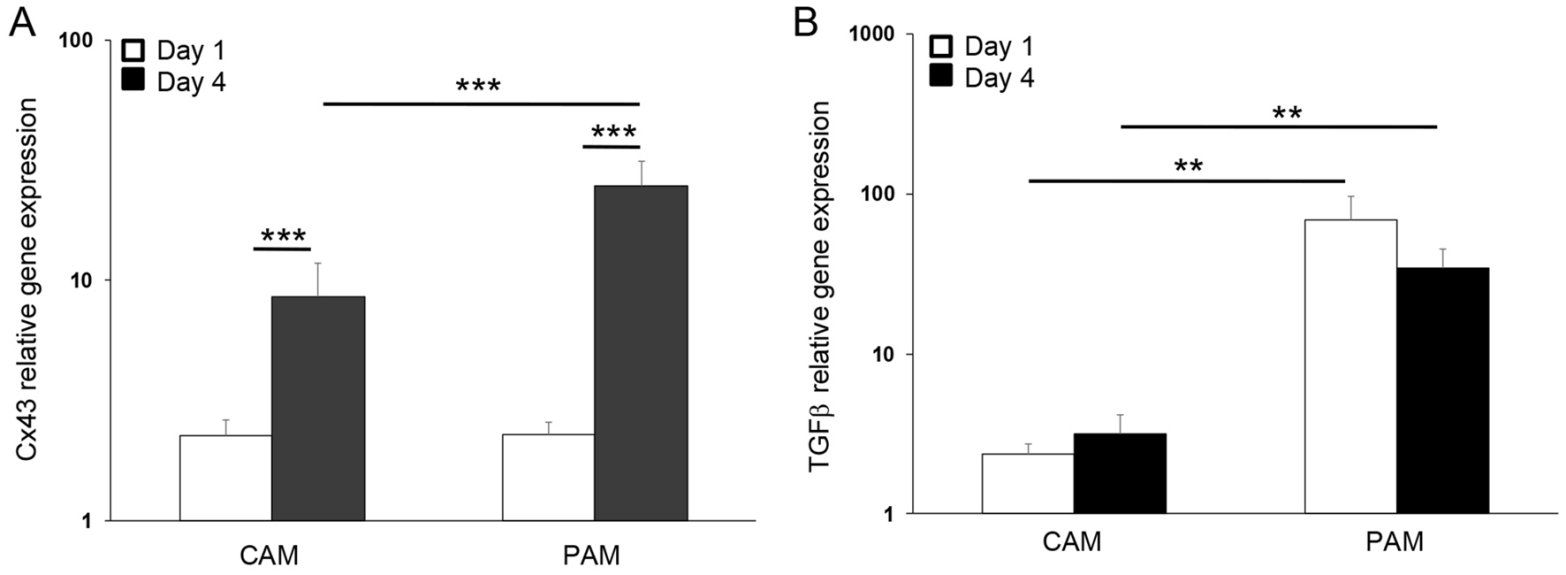
The effect of trauma on Cx43 and TGFβ_1_ gene expression. Term human CAM and PAM specimens were cultured for up to four days after trauma. Gene expression of Cx43 (**A**) and TGFβ_1_ (**B**) were presented as ratio values and normalised to Day 1 controls. In all cases, error bars represent the mean and SEM values of 9 replicates from three separate donors, where (*, ** or ***) indicates significant comparisons for CAM or PAM specimens. All other comparisons (not indicated) were not significantly different.

Figure 8 examines the effects of trauma on Cx43 and TGFβ1 gene expression. At day 1, no significant differences were found for Cx43 gene expression in CAM and PAM specimens. After 4 days, trauma increased Cx43 gene expression, with values significantly greater for PAM than CAM specimens (all p < 0.001; Fig. 8A). Whilst TGFβ1 gene expression was higher for PAM than CAM specimens (both p < 0.01; Fig. 8B), we found no significant differences when comparing time points after trauma.

## Discussion

Cx43 regulates distinctive wound healing mechanisms in AM defects resulting in cell adhesion, proliferation and migration at different stages of the healing process. Acceleration of wound healing is dependent on the mobility dynamics of Cx43 delivery to the cell membrane elevating inflammatory cytokines via gap junction communication or hemichannel cluster formation to form large Cx43 plaques^22–27^. In wounded skin tissues, cells that were found to promote Cx43 plaque formation localized to the plasma membrane are well known to increase cell adhesion leading to reduced migration, apoptosis and loss of wound re-epithelialisation^23, 25, 26^. In AM defects, the abundance of Cx43 is strongly increased by AMCs and has been previously reported to affect wound healing outcomes in chronic skin wounds and AM wounds^22–25^. However, it is possible that the expression and function of Cx43 is influenced by distinctive phenotypic properties of AM cell populations and may contribute to the differential healing mechanisms in wounded AM defects. The present study characterized the morphological differences in healing potential of AM cell populations after trauma.

We used a double immunostaining confocal imaging technique to detect αSMA expressing myofibroblasts with an antibody that recognized the Cx43 molecule localized most abundantly in the long cytoplasmic cellular processes. Intracellular Cx43 localization was not found for vimentin-positive AMCs which showed abundant plaque-like structures between cell-to-cell contacts in the fibroblast layer close to the wound edge. The sub-cellular dynamic localization of Cx43 staining in the cytoplasm or nucleus is well known to have a differential effect on transcriptional activity and influence the efficiency adhesion or cell migration. Whilst the present study did not quantify the rate of cell migration into the defect site using 2D monolayer models after cell expansion, incomplete or poor healing mechanisms have previously been attributed to the abundance at the cell-to-cell contacts as Cx43 plaques. For example in chronic wounds such as diabetic, ulcers or skin injuries, the overexpression of Cx43 leads to phenotypic changes in fibroblasts and keratinocyte cell populations that mediate an inflammatory response without wound closure^25–27^. In contrast, the downregulation of Cx43 with antisense oligonucleotides in skin wounds accelerated healing by increased TGFβ1 expression that induced an activation myofibroblast phenotype with elongated lamellopodia^26, 28^. In corneal endothelial injuries, Cx43 expression increased enabling fibroblast contraction and activation of pro-inflammatory pathways, whilst knockdown of Cx43 prevents αSMA myofibroblast activation^29^. In fetal membranes, the link between Cx43 expression and healing may be influenced by the spatial–temporal dynamics and localization of Cx43 leading to differential effects on cell populations that could hinder complete closure of the wound. Whilst we observed the presence of myofibroblasts but not AMCs in the defect site as shown by time-lapse imaging [Supplementary Video], this mechanism is dependent on the concentration and localisation of Cx43. In the present study, the levels of Cx43 expression by AMCs increased at the WE, enabling differentiation to myofibroblasts, activation of a pro-inflammatory mechanism to promote wound contraction. We also observed macrophages around the defect site which may have been recruited from the amniotic fluid after culture and stimulate AMC differentiation into myofibroblasts after αSMA and TGFβ activation, as described by others.^7^ However, as plaque formation in AMCs increased with culture in amniotic fluid samples, Cx43 overexpression slowed down complete closure of the wound. This will be dependent on the model system targeted by pharmacological agents to knockdown Cx43, since gene expression and wound healing will be different in preterm membranes subjected to mechanical stimulation and warrants further investigation involving mechanotransduction studies.

Using SHG imaging, the present study demonstrates highly polarized collagen fibres within the fibroblast layers during the wound healing process. The mechanical properties of the AM have been characterized by several research groups, with collagen presence playing a key role in maintaining mechanical integrity of the tissue^30–33^. Loss of collagen deposition and changes in alignment after mechanical stimulation alters elastin and collagen release, influencing downstream proinflammatory signalling pathways^30, 31^. The small fetal defects exhibit characteristics of purse-string contraction healing, a mechanism previously seen in embryonic wound healing^20, 21, 34, 35^. Cell traction forces by myofibroblasts are generated through actomyosin filament sliding, causing cells to move through the ECM via focal adhesions^36, 37^. The findings from the present study are concurrent to the increase in density of αSMA expressed by myofibroblasts in the fibroblast and reticular layers of the fetal membranes taken from patients undergoing different labour inductions^38^. The enhanced cellular activities by AMCs and myofibroblast cell populations contributed to an increase in tissue thickness after fetal membrane rupture. AMCs differentiation and activation of pro-inflammatory signalling during fetal membrane rupture and/or pathogen exposure is well known to promote tissue remodelling^15, 39^. Indeed, αSMA expression increased contractility of myofibroblasts by modifying these myosin stress fibres, particularly when treated with TGFβ1.^15^ In small mouse AM defects, AEC to AMC transition took place by AECs and this population was found to migrate from the epithelial layer across the defect site while the fibroblast layer remained compromised^7^. The reversible transition of AECs and AMCs during pregnancy and labour involved changes in vimentin expression by AMCs as a marker to characterise this process^7, 38, 39^. Interestingly, damage caused by cigarette exposure of AM explants or cell populations increased expression of vimentin and N-cadherin leading to a greater transition between cell states triggered by TGFβ^39, 40^. In embryonic or skin wound healing models, fibroblasts and myofibroblast are well known to have distinct phenotypical characteristics^20, 21, 34, 35, 41^. Fibroblasts are spindle-shaped, express vimentin, but not αSMA. Myofibroblasts express αSMA and have elongated cytoplasmic structures distinctive from fibroblasts, including extensive actin stress fibers. Cell–matrix adhesions, abundant intercellular adherens and Cx43^17, 19, 42, 43^. Thus, inflammation induced by trauma or oxidative stress will differentially influence cell behaviour in a sub-population dependent manner and further highlights the need for better human models to understand wound healing in the AM.

In summary, we showed evidence of potential locomotive AM cell populations dependent on Cx43 localisation. Whilst AMCs expressed Cx43 plaques, we observed the presence of myofibroblasts in the defect site with Cx43 localised in the cytoplasm. Myofibroblasts synthesise collagen fibres in the defect site and begin to pull the edges of the AM wound by collagen contraction. This mechanism is influenced by TGFβ1 and should be explored further utilising human biomechanical defect models. Successful repair of the membranes could promote tissue remodelling mechanisms that restore biomechanical function, reduce the risk of tissue contraction, chorionic membrane separation, amniotic fluid leakage, subsequent uterine infection and preterm birth.

## Methods

All methods were performed according to the relevant guidelines and regulations at University College London Hospital and the School of Engineering and Materials Science, Queen Mary University of London. Ethical approval for collection of amniotic fluid and fetal membrane samples was granted by the Joint University College London and University College London Hospital Committees, the National Research Ethics Service Committee London, Bloomsbury and the Ethics of Human Research Central Office (REC reference 14/LO/0863). All patients gave written informed consent to provide samples before procedures were performed. The methods are well established and have been described previously^24, 30, 31^.

### Amniotic membrane tissue isolation and amniotic fluid sample collection

Term human fetal placentas and amniotic fluid were collected from each donor (n = 6, mean GA = 38 weeks; mean maternal age = 35 years) with written informed consent from women undergoing delivery by elective caesarean section at University College London Hospital. Women in spontaneous labour or with placenta praevia, multiple pregnancy, antepartum haemorrhage, ruptured membranes, pre-eclampsia or small for gestational age were excluded. At caesarean section before delivery of the placenta, a sterile Babcock tissue clip was placed on the lower edge of the FM within the uterine incision to provide a landmark of the region close to the cervix. The placenta was separated from the uterus and the FM by gentle traction and rinsed with Earle’s Balanced Salt Solution (EBSS). The AM was separated from the CM and the specimens dissected into 20 × 20 mm explants from tissue regions close to the cervix (CAM) or placenta (PAM). All specimens were washed with EBSS and cultured in human amniotic fluid samples from patient matched donors.

A proposed consensus nomenclature for cell sources isolated from AM tissue explants with distinction between AECs and human amniotic mesenchymal stromal cells (hAMSCs) has been presented recently^44^. On further examination, hAMSCs were isolated in the AM after collagenase digestion and this population may be different to differentiating and proliferating AMCs and myofibroblasts found in situ and are embedded within a 3D collagen matrix environment^45^. Whilst we recognise the differences in the terminology used to identify a variety of cell sources isolated from AM for applications in cell therapy, hAMCs extracted from AM explants as a total population after collagenase digestion are not the same as differentiating AMCs and myofibroblasts detected in situ using novel multiphoton confocal and SHG imaging techniques. The present study uses the terminology of AECs, AMCs and myofibroblasts similar to previous studies^2, 7, 15, 24, 30, 31, 38–40^.

### Effects of culture of AM explants with amniotic fluid samples after trauma

The present study utilised a well-established in vitro AM defect model to examine the effects of trauma^24^. A 21 Gauge needle with a wall thickness of 0.15 mm was used to create a 0.8 mm diameter equivalent to that created by an amniocentesis intervention in CAM and PAM specimens. The tissue explants were secured within the CellCrown insert with a size of 5 mm diameter in a 24 well plate. Amniotic fluid samples (2 ml) were injected into the well below the specimen and cultured for up to 4 days. All amniotic fluid samples were taken from the same donor as the tissue explants. At the end of the culture period, control or wounded CAM or PAM specimens were either fixed in 4% PFA or stored in RNA Later at − 20 °C prior to analysis.

### Immunostaining

Whole-mount control and wounded specimens were fixed in 4% PFA for 2 h and incubated with primary antibodies for mouse Cx43 (diluted 1:100, ThermoFisher Scientific, CX-1B1), rabbit vimentin (1:500, Abcam, ab137321) or rabbit αSMA (1:100, Abcam, ab5694) at 4 °C overnight, as previously described^24, 30^. Specimens were washed with PBS and incubated with Alexa-Fluor 568 anti-mouse IgG or Alexa Fluor 488 Goat anti-rabbit IgG secondary antibody for 2 h at room temperature (1:1000, Life Technologies) and counterstained with 1 μg/ml of the nuclear dye DAPI for 20 min (1:1000). Secondary antibody incubation in the absence of the primary antibody was used as a negative control.

### Confocal microscopy and SHG imaging

Control and wounded CAM and PAM specimens were imaged in the epithelial layer and compared to the fibroblast layer using two photon confocal imaging on a Leica SP8 with a Coherent Chameleon Ultra, Ti Sapphire mode locked IR laser (Leica, Milton Keynes, UK), as previously described^24, 31^. Samples were imaged at excitation/emission wavelengths of 405/460 nm for DAPI, 495/518 nm for vimentin or αSMA and 578/603 nm for Cx43. A transmission detector was used for the collection of collagen SHG signal with a 430 to 450 nm barrier filter with a pump wavelength of 880 nm at 80 fs pulse width. A constant step size (Z-section interval) of 1.5 μm was used across all Z-stack images collected. All parameters including detector gain, offset and laser power were kept constant to enable quantification. Images were processed using ImageJ software (version win 64) or Imaris 9.5.0. High resolution images were taken on the Zeiss LSM 980 with Airyscan 2 microscope (Zeiss, Oberkochen, Germany) with × 42 magnification and identical excitation/emission wavelengths for DAPI and αSMA.

### Confocal image quantification

Cx43 levels were quantitatively evaluated per tissue area and per cell nuclei in control and wounded CAM or PAM specimens, using a well-established pixel-counting method, using well-established methods as previously described^24^. Maximum projections were performed in the AM to compare cell populations in the epithelial and fibroblast layer. The images were converted to binary using identical threshold values and objects exceeding 2 pixels were counted to identify Cx43 positive pixels per tissue area (500 μm^2^) or myofibroblast nuclei (Supplementary Fig. S1). In separate experiments, αSMA quantification compared CAM and PAM specimens in the fibroblast layer only by Imaris 9.5 using the volume and surface commands (Supplementary Fig. S2). Pixels were counted to identify αSMA positive pixels per tissue area (500 μm^2^) or the volume of αSMA expressed by mesenchymal cells (μm^3^) and Cx43 plaques compared (Supplementary Fig. S3). Nuclei deformation was quantified by measuring the circularity (4π × area/perimeter^2^) of each nucleus where a value of 1.0 indicates a perfect circle or value of 0 indicates an increasingly elongated shape using the particle analysis method (Image J).

### SHG quantification

SHG intensity was characterized by plotting 3D surface plots to compare collagen distribution in the epithelial and fibroblast layer in control and wounded specimens, as previously described^24, 31^. To characterize the direction of collagen alignment, an orientation distribution analysis using the Directionality ImageJ plug-in (v2) was performed, using well-established methods^24, 31^. After binary conversion, the SHG images were subjected to 2D distribution analysis using the local gradient orientation method, as described^24, 31^. The plug-in calculated the number of objects that distribute between 0° and 180° with a bin size of 1°.

### Scanning electron microscopy

The structural morphology of the cell sub-populations in fetal membrane explants was analysed by Scanning Electron Microscopy (SEM) (Supplementary Fig. S4). After trauma, human FM specimens were fixed in 4% paraformaldehyde for 2 h at room temperature, washed with Milli-Q water, dehydrated with ethanol solutions (20%, 50%, 70%, 90%, 96%, and 100% v/v) for 10 min each and dried to critical point (K850, Quorum Technologies, UK). The specimens were mounted on 10 mm mounting block, sputter coated with 10 nm gold particles and analysed on the FEI Inspect F50 (FEI Comp, The Netherlands).

### Real-time qPCR

Total RNA was extracted from AM specimens with Trizol reagent and purified using RNeasy Mini Kit (Qiagen, Manchester, UK), as previously described^31^. Briefly, RNA (200 ng) was reverse transcribed with Enhanced Avian RT First Strand cDNA synthesis and oligo(dT) 23 primer (Sigma Genosys, Cambridge, UK). The qPCR samples were run in triplicate containing 5 μl SYBR green mastermix, 2.5 μl cDNA and 2.5 μl primer pair sequences for Cx43 sense: 5′-CTCGCCTATGTCTCCTCCTG-3′, antisense: 5′-TTGCTCACTTGCTTGCTTGT-3′, TGFβ1 sense: 5′-CCCAGCATCTGCAAAGCTC-3′ and antisense: 5′-GTCAATGTACAGCTGCCGCA-3′ and GAPDH sense: 5′-TCTCTGCTCCTCCTGTTC-3′, GAPDH antisense: 5′-CGCCCAATACGACCAAAT-3′. PCR products were detected on the StepOnePlus Real-Time PCR System (ThermoFisher Scientifc), as previously described.^31^ Relative gene quantification of Cx43 or TGFβ1 was calculated by normalizing the target to the reference gene, GAPDH and to the calibrator sample by a comparative Ct approach and ratio values expressed on a logarithmic scale.

### Statistical analysis

All values are expressed as the mean and ± SEM. Statistical analysis was performed by a two-way analysis of variance (ANOVA) and the multiple post hoc Bonferroni corrected t-tests to compare differences between controls (no defect) and traumatised groups in CAM and PAM specimens. In all cases, values of p < 0.05 were considered statistically significant. The number of replicates for each test condition from separate donors are indicated in the figure legend.

## Supplementary Information

Supplementary Information 1.

Supplementary Video 1.
