# Supplementary material for: Cx43 mediates changes in myofibroblast contraction and collagen release in human amniotic membrane defects after trauma
Source: Sci Rep. 2021 Aug 18;11:16975. doi: 10.1038/s41598-021-94767-4 (PMC8373966; doi:10.1038/s41598-021-94767-4)
Supplement: Supplementary file 1 — Supplementary Information 1. [file 41598_2021_94767_MOESM1_ESM.pdf]

Cx43 mediates changes in myofibroblast contraction and collagen release in human amniotic membrane defects after trauma

Eleni Costa[1], Babatunde O. Okesola[1], Christopher Thrasivoulou[2], David L. Becker[3], Jan A. Deprest[4,5], Anna L. David[4,5], Tina T. Chowdhury[1,*]

[1]Institute of Bioengineering, School of Engineering and Materials Science, Queen Mary University of London, Mile End Road, London E1 4NS, UK.

[2]Department of Cell and Developmental Biology, University College London, Gower Street, London. WC1E 6BT, UK.

[3]Lee Kong Chian School of Medicine, Nanyang Technological University, 11 Mandalay Road, Singapore.

[4]Department of Obstetrics and Gynecology, University Hospitals Leuven, Leuven, Belgium.

[5]Institute for Women's Health, University College London, Medical School Building, Huntley Street, London WC1E 6AU, UK.

[1*]Corresponding author

Dr Tina Chowdhury. Institute of Bioengineering, School of Engineering and Materials Science, Queen Mary University of London, Mile End Road, London E1 4NS, UK.

Email address: t.t.chowdhury@qmul.ac.uk

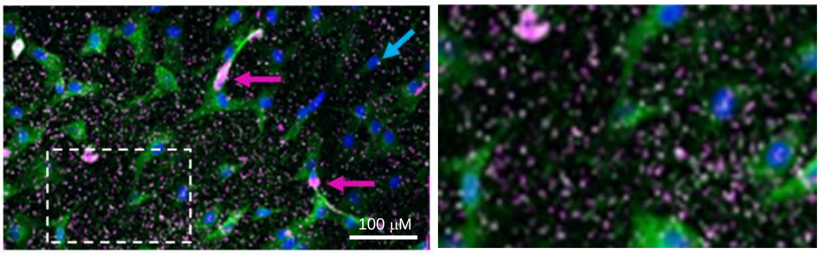

Cx43 pixel counting

1. Max projection
2. Thresholding
3. Binary conversion
4. Particle analysis in cytoplasm.
5. Remove <2 pixels [background]

Cx43 protein distribution per cell type (*pixels per cell nuclei)*							
Amniotic epithelial cells (AECs); Amniotic mesenchymal cells (AMCs); Myofibroblast (MFs). Values represent ±SEM = 9 replicates from three donors.		CAM			PAM		
		AECs	AMCs	MFs	AECs	AMCs	MFs
Day 1	Control	0.7 (±0.09)	4.6 (±1.67)	10.7 (±1.03)	0.7 (±0.02)	8.9 (±1.57)	17.5 (±0.42)
	WE	2.2 (±0.27)	17.8 (±1.45)	37.3 (±0.28)	2.7 (±0.07)	9.5 (±1.3)	36.8 (±0.65)
Day 4	Control	0.6 (±0.05)	13.1 (±1.52)	10.3 (±1.01)	0.7 (±0.03)	15.4 (±1.96)	16.9 (±0.32)
	WE	4.7 (±0.15)	20.8 (±1.46)	47.4 (±1.59)	5.0 (±0.08)	28.6 (±1.69)	49.9 (±0.24)

Supplementary Fig. S1. Analysis of Cx43 protein plaques in the nuclei and cytoplasm of human cell sub-populations present in the amniotic membrane. The method of Cx43 pixel counting was analysed with ImageJ (Fiji) software as previously described [24, 30]. Top panel shows particles outside the cytoplasmic borders were excluded for counting. All images were converted to 8-bit black and white using identical threshold values. Images were then transformed to binary (light background) with a limit of 2 pixels to Infinity to identify Cx43 positive pixels and to ensure any background noise of w pixel units and lower was removed. The analyse particle measurement was applied, yielding results in pixels. After counting, Cx43 plaques were seen as outlines to ensure counting within cytoplasm. Cx43 protein expression formed 0.1 mm to 1 µm plaques with varying sizes and shapes within the nuclei and cytoplasmic bodies. Maximum projections of the fibroblast and epithelial layers in the amniotic membrane were used for identification of the cell sub-populations. Data was normalised per cell nuclei (pixels) or per tissue area (um^2) and the differences between AECs and MFs are shown in Fig. 5. The values for Cx43 protein distribution per cell type are shown in the bottom panel.

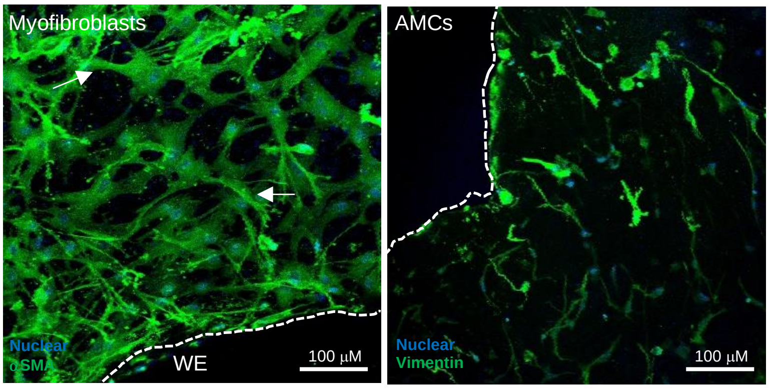

Cell volume/tissue area (μm^3)		CAM		PAM	
		AMCs	MFs	AMCs	MFs
Day 1	Control	14.2 (±2.1)	6.5 (±0.2)	11.7 (±2.3)	3.45 (±0.2)
	WE	20.2 (±2.4)	46.1 (±3.4)	18.7 (±0.8)	32.2 (±1.3)
Day 4	Control	19.8 (±2.6)	18.7 (±2.7)	20.1 (±2.5)	5.0 (±0.1)
	WE	35.3 (±6.7)	95.6 (±3.2)	43.6 (±1.3)	66.4 (±3.4)

Values represent ±SEM for 9 replicates from three donors.

Supplementary Fig. S2. Cell volume differences in myofibroblasts and AMCs. Human amniotic membranes were immunostained with either αSMA or vimentin to identify myofibroblasts and AMCs respectively by confocal microscopy. We observed highly elongated morphology for both cell types. However, myofibroblasts expressed αSMA and showed the presence of lamellipodia structures that extend out of the cytoplasmic processes as indicated by the white arrows (Top Panel). In contrast. we observed these structures at a reduced amount in AMCs which expressed vimentin, as characterised by cell volume per tissue area (Bottom Panel).

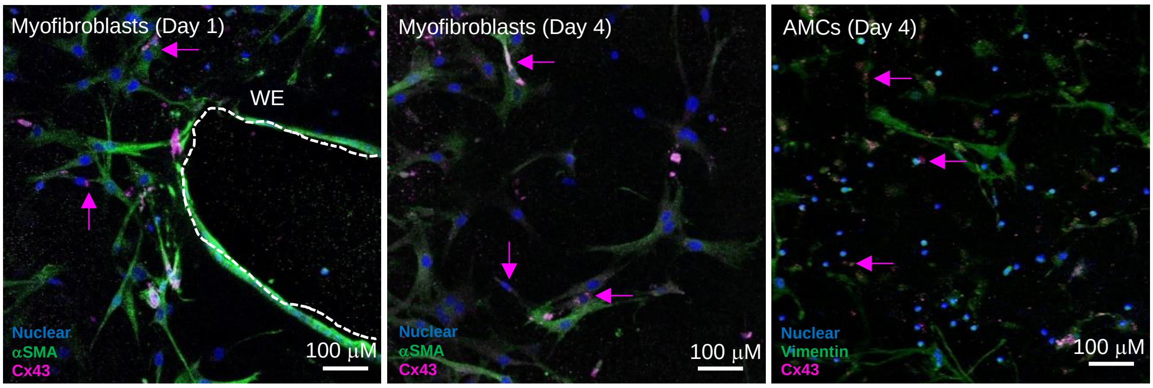

Supplementary Fig. S3. Comparison of Cx43 protein plaque formation in myofibroblasts and AMCs present in the fibroblast layer of the human amniotic membrane after trauma. We observed high levels of Cx43 protein expression in the cytoplasm of myofibroblasts (pink arrows) after 1 or 4 days of trauma. In contrast, AMCs showed Cx43 localisation between cytoplasmic bodies at cell to cell contacts.

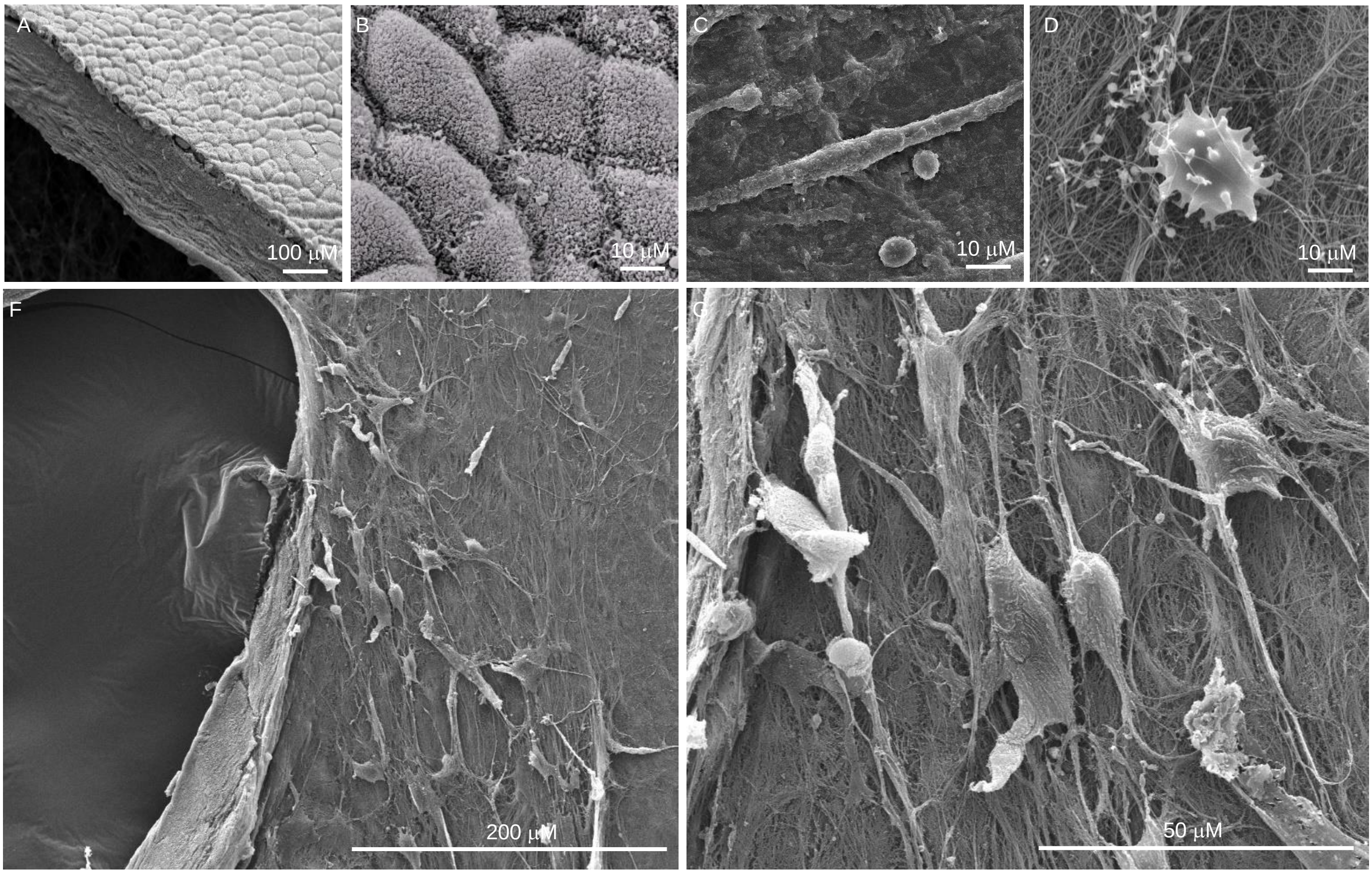

Supplementary Fig. S4. Analysis by SEM showed structural differences in the cell sub-populations present in the human fetal membranes. Tissue explants from fetal membranes showing a flattened blanket of AECs in the epithelial layer of the AM (A). High magnification shows cuboidal structure of the AECs (B), elongated structure of AMCs (C), macrophages (D) and highly polarised cytoplasmic bodies of myofibroblasts around the edges of the AM wound (F, G). White bars indicate magnification for each SEM image.

Supplementary Video. Mechanism of cell contraction, migration and wound closure in AM defects. After trauma, we observed increased cellularity around the wound edge (WE) and the presence of αSMA expressing myofibroblasts that had migrated into the defect site and released collagen. AECs formed a thick layer around the WE but this cell population did no not migrate into the defect site in the epithelial layer. AMCs express vimentin (green) in the stress fibres that increased cytoplasmic and nuclear contraction, but the cells did not migrate into the defect site. We observed punctate staining of Cx43 expression (pink) by myofibroblasts with nuclear and cytoplasmic localization after trauma. In the fibroblast layer, myofibroblast migrate into the wound area and synthesise collagen fibres. The increased cellularity by vimentin positive AMCs (green) and myofibroblast result in collagen contraction and remodelling across the AM defect site (D). Blue (nuclear), green (αSMA), pink (Cx43) and red signals (collagen) were detected by confocal microscopy and SHG imaging, respectively. Scale bar = 50 μM.
