# Supplementary figures and images for: Cx43 mediates changes in myofibroblast contraction and collagen release in human amniotic membrane defects after trauma
Source: Sci Rep. 2021 Aug 18;11:16975. doi: 10.1038/s41598-021-94767-4 (PMC8373966; doi:10.1038/s41598-021-94767-4)

## Slide 1
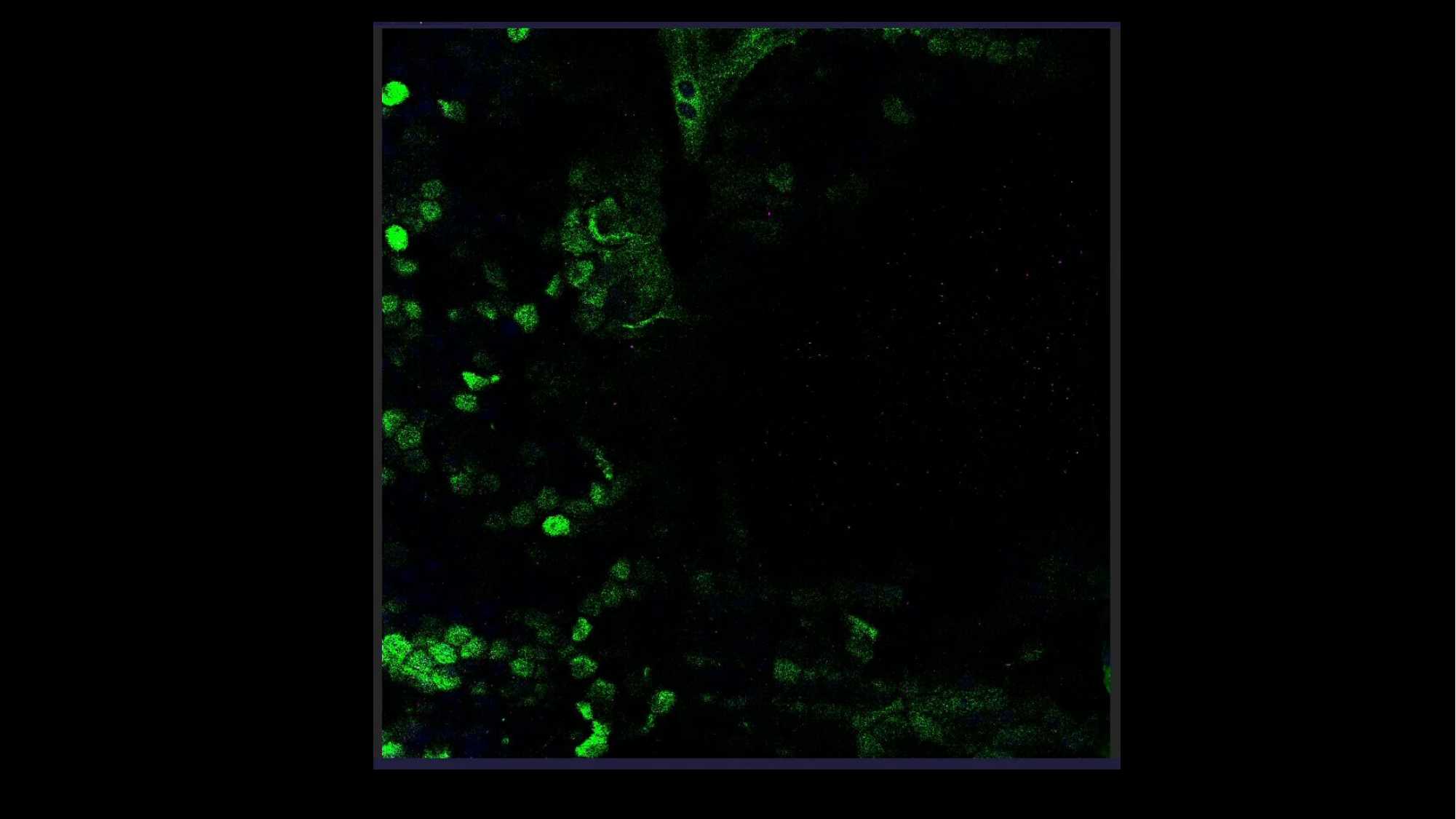

Supplement: Supplementary file 2 — Supplementary Video 1. [file 41598_2021_94767_MOESM2_ESM.pptx]
